# Spectral analysis combined with advanced linear unmixing allows for histolocalization of phenolics in leaves of coffee trees

**DOI:** 10.3389/fpls.2014.00039

**Published:** 2014-02-18

**Authors:** Geneviève Conéjéro, Michel Noirot, Pascale Talamond, Jean-Luc Verdeil

**Affiliations:** ^1^Plant Cell Imaging platform PHIV UMR AGAP (Cirad, SupAgro, INRA), UMR B&PMP (INRA, CNRS, UM2, SupAgro)Montpellier, France; ^2^UMR PVBMT, (CIrad, IRD) La RéunionFrance; ^3^Institut des Sciences de l'Evolution, UMR ISE-M (CNRS, IRD, UM2)Montpellier, France

**Keywords:** spectral analysis, multiphotonic microscope, autofluorescence, caffeoylquinic acids, xanthone, *Coffea*

## Abstract

An imaging method using spectral analysis combined with advanced linear unmixing was used to allow histolocalization of natural autofluorescent compounds such as hydroxycinnamic acid (chlorogenic acid) and xanthone (mangiferin) in living cells and tissues (mature coffee leaves). The tested method included three complementary steps: 1/ visualization of natural autofluorescence and spectrum acquisition with a multiphoton microscope; 2/ identification of some compounds using previous information on the chemical composition of the tissue, obtained from litterature; and 3/ localization of candidate compounds by spectral imaging. The second part of the study consisted of describing the histochemical structure of leaves during their development. This revealed very fast histochemical differentiation of leaves during the first week after their emergence. Lastly, young leaves of *Coffea pseudozanguebariae* (PSE), *C. eugenioides* (EUG), *C. arabica* (ARA) and *C. canephora* (CAN) were compared. This confirmed the presence of xanthone in PSE and EUG, but especially its precise tissue localization. This also highlighted the paternal CAN origin of the leaf structure in the allotetraploid species ARA. The limits and advantages of the method without staining are discussed relative to classical epifluorescence microscopy under UV light. This non-invasive optical technique does not require pretreatment and is an effective experimental tool to differentiate multiple naturally-occuring fluorochores in living tissues.

## Introduction

Plants produce a vast array of secondary metabolites such as phenolics that are estimated to comprise at least 8000 different chemicals (Jones et al., 2013). Many of them include low molecular weight phenolics, but also condensation products such as lignins and flavonoids. It is estimated that phenolics represent about 40% of all organic compounds circulating in the biosphere. These contribute to hardiness, color, taste, odor and many of them are of high economic value.

In the *Coffea* genus, caffeoylquinic acids (CQA) and dicaffeoylquinic acids (diCQA)-two secondary metabolites from the phenylpropanoid pathway-are of major economic importance in coffee production via two major species: *Coffea arabica* L. (ARA) (65–70% of the worldwide coffee production) and *C. canephora* Pierre ex Froehner (CAN). CQA and diCQA are indeed the most abundant soluble hydroxycinnamic acids (HQA) present in leaves and seeds (Clifford, 1985; Ky et al., 2001; Campa et al., 2012). Three isomers occur in each class according to the acylating residue positions, but the most important in terms of content is 5-*O*-caffeoylquinic acid, more commonly known as chlorogenic acid (Clifford, 1985) (see Figure A in supplementary material).

Another important xanthonoid phenol has been identified in leaves of *C. pseudozanguebariae* Bridson (PSE). This is *C*-glycosyl xanthone, or 2-*C*-β-D-glucopyranosyl–1,3,5,7 tetrahydroxyxanthen-9-one, more commonly known as mangiferin (Talamond et al., 2008, 2011; Campa et al., 2012) (see Figure A in supplementary material). As for chlorogenic acid (Bertrand et al., 2003; Mondolot et al., 2006), its content was found to decrease from young to mature leaves (Campa et al., 2012). Lastly, it has also been detected in other *Coffea* species such as *C. eugenioides* S. Moore (EUG) and ARA, but is absent in CAN leaves (Campa et al., 2012).

Great progress has been made in understanding the regulation of the expression of genes involved in phenol metabolism, but less is known about their spatial distribution at the tissular and cellular level. To date, phenol histolocalization is generally done using Neu's reagent, which binds with phenolics, emitting a specific greenish-white epifluorescence under UV light (Neu, 1956). As expected, the most intense greenish-white fluorescence is observed in juvenile coffee leaf blades (Mondolot et al., 2006; Campa et al., 2012), but its specificity toward HQA is low. By contrast, mangiferin histolocalization by epifluorescence does not require any reagents and has been directly observed through its autofluorescence (Talamond et al., 2011; Campa et al., 2012). As phenolics are autofluorescent compounds, it was suggested that microspectrometry allowing fluorescence spectra recording from ROI could be used to confirm their fluorescence signature (Hutzler et al., 1998). This can be achieved using either confocal (Hutzler et al., 1998) or two-photon microscopy (Talamond et al., 2011). The latter has the advantage of providing excellent three-dimensional spatial resolution of the location from which the fluorescence spectra can be obtained (Saadi et al., 1998). This imaging approach is not commonly used to localize phenolic compounds since fluorescence spectra obtained from plant samples are generated by the emission of many fluorophores with overlapping emission peaks. Fortunately, spectral unmixing methods using computer algorithms provide an opportunity to separate a spectral signal recorded from a single or a group of pixels containing a number of fluorophores into separate intensity signals of each of them.

In this paper, a new imaging approach was carried out to localize phenols *in situ* using a multiphoton microscope combined with spectral analysis and linear unmixing. This allows the differentiation of distinct fluorophores with highly overlapping emission spectra (Zimmermann et al., 2003; Garini et al., 2006). We developed such a method to localize 5-CQA and mangiferin, which are fluorescent compounds, in fresh mature coffee leaves. Nevertheless, it is important to choose plant material with a high proportion of 5-CQA in HQAs in order to minimize noise due to other HQAs. As the highest CQA/HQA ratio (93%) was found to characterize mature ARA leaves (estimated from Campa et al., 2012), we opted to use this material in the first part of the present study. The second part involved describing the differentiation of the histochemical structure of ARA leaves over time. Lastly, the histochemical structure of young leaves was compared between four *Coffea* species, i.e., PSE, EUG, ARA, and CAN. PSE was selected for its very low 5-CQA content (Bertrand et al., 2003) and high mangiferin content in leaves (Talamond et al., 2011; Campa et al., 2012). EUG and CAN were taken into account because they are putative parents of ARA (Lashermes et al., 1999). In addition, the four species constitutes a gradient for the chlorogenic acid content (Campa et al., 2012).

## Materials and methods

### Plant material

For *Coffea pseudozanguebariae* (PSE), *C. eugenioides* (EUG), *C. arabica* L (cv Bourbon, ARA) and *C. canephora* (CAN), leaves were sampled on coffee trees growing in the field at St Pierre, Réunion (France). Mature leaves were sampled on the third node from the branch tips, whereas young leaves were sampled at phase φ2 and φ3 (Lécolier et al., 2009).

### Histological and imaging methods

Leaf cross-sections (50 μm) were obtained using an HM 650 V vibrating blade microtome (Microm, Waldorf, Germany) and then dipped in a glycerol/water (50:50) solution saturated with ascorbic acid to prevent oxidation. A multiphoton Zeiss 510 META NLO microscope (Zeiss, Iena, Germany), equipped with a laser Chameleon Ultra II Ti-Sapphire (Coherent, Santa Clara, California) and a 25x/0.8 Plan-Neofluar immersion objective, was used to obtain galleries of spectral images and emission spectra from fresh leaf sections. Spectral imaging was carried out with laser optimal excitation at 720 nm, reproducing laser UV monophotonic excitation in the 365–700 nm emission range. A set of 32 images was obtained, with each image being acquired with a separate narrow bandwidth (10.7 nm), representing the complete spectral distribution of the fluorescence signals for every point of the microscopic image. This procedure was performed on leaf cross-sections, as well as on pure chlorogenic acid (5-CQA) and mangiferin powders (Sigma-Aldrich, St Quentin Fallavier, France and Extrasynthese, Genay, France, respectively).

To check a possible impact of pH on spectral emission, some experiments using different pH (between pH = 5.5 and 10.5) were carried out in the case of the 5-CQA. There was no impact on the results (data not shown).

The spectral analysis was carried out using the advanced linear unmixing function (LSM 510 software) which separates mixed signals pixel by pixel using the entire emission spectrum of each defined autofluorescent compound in the sample. This function requires at least two spectra and was applied with the advanced iterative option and one residual channel. After spectral imaging acquisitions on leaf cross-sections, the advanced linear unmixing function allowed visualization with coded colors of the fluorescence of chlorogenic acid (5-CQA), mangiferin, and chlorophyll in cells based on their reference spectra.

Each experiment was repeated with 3–5 leaves per different stages or species.

## Results

### Characterization of leaf anatomy under fluorescence

**Figure 1:**
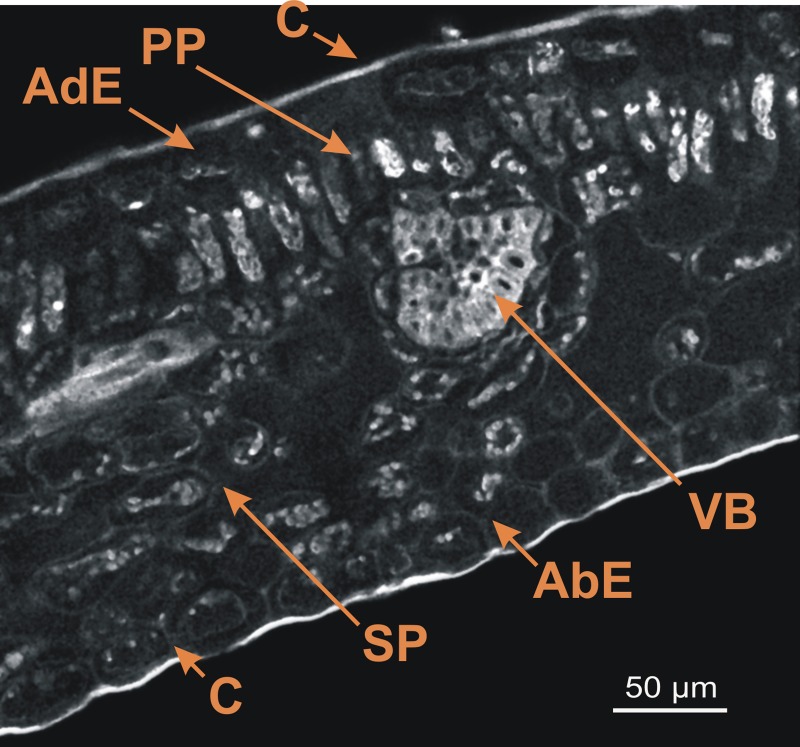
**Spectral image on leaf cross-section of the *C. arabica* cv. Bourbon obtained using a multiphoton microscope at 720 nm excitation**. AdE, adaxial epidermis; AbE, abaxial epidermis; C. cuticule; PP, palisade parenchyma; SP, spongy parenchyma; VB, vascular bundles. Scale bar = 50 μm.

The first step involved obtaining spectral images from mature leaf cross-sections of the ARA (*C. arabica* L cv Bourbon) (Figure 1). This image was the result of merging 32 images (channels) obtained between 365 and 700 nm and shows the whole fluorescence detectable in the leaf section without staining. The autofluorescence of cell walls, pigments and cuticle enabled visualization of the leaf anatomical structure.

As described by Dedecca (1957), leaf cross-sections showed the classical foliar structure with the adaxial epidermis, palisade parenchyma, spongy parenchyma, and abaxial epidermis. Each epidermis was covered by a thin cuticle. Lastly, vascular bundles were also present on this cross-section.

**Figure 2:**
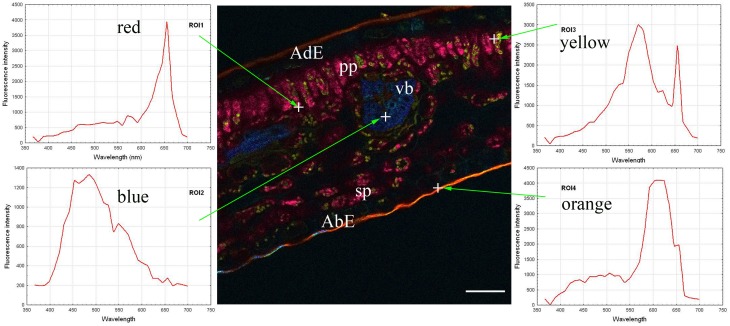
**Coded autofluorescence in a *C. arabica* cv. Bourbon leaf blade cross-section observed using a multiphoton microscope at 720 nm excitation**. Four spectra emissions were recorded, corresponding to red (ROI1), blue (ROI2), yellow (ROI3) and orange (ROI4) colors, respectively on chloroplasts, vascular bundle, unknow cellular structure and cuticle. AdE, adaxial epidermis; AbE, abaxial epidermis; C, cuticule; PP, palisade parenchyma; SP, spongy parenchyma; VB, vascular bundles. Scale bar = 50 μm

The autofluorescence software coding function was then applied, thus generating the central part of Figure 2. Coding consisted of giving a wavelength-dependent color to each pixel with a proportional intensity to the pixel fluorescence intensity (Garini et al., 2006). This encoding clearly brought out the previously described leaf structure (Figure 1). Three groups of tissues were distinguished according to the autofluorescence intensity and color: 1/both epidermal tissues (adaxial and abaxial), which had low fluorescence; 2/parenchyma (palisade and spongy) was characterized by strong red fluorescence; and 3/vascular bundles showing strong blue fluorescence. Cuticles also showed high orange fluorescence. Lastly some small yellow fluorescent zones were observed within the palisade parenchyma.

The next step consisted of obtaining spectra from red, blue, yellow and orange zones (on the left and right parts of Figure 2). In the present case, each spectrum was characteristic of one pixel, symbolized by a cross on the figure, but it was also possible to define each emission spectrum from a small number of pixels, giving identical results. The next step involved looking for compounds that could explain the autofluorescence of these different ROI.

### Identification and localization of 5-CQA, mangiferin and chlorophyll

**Figure 3:**
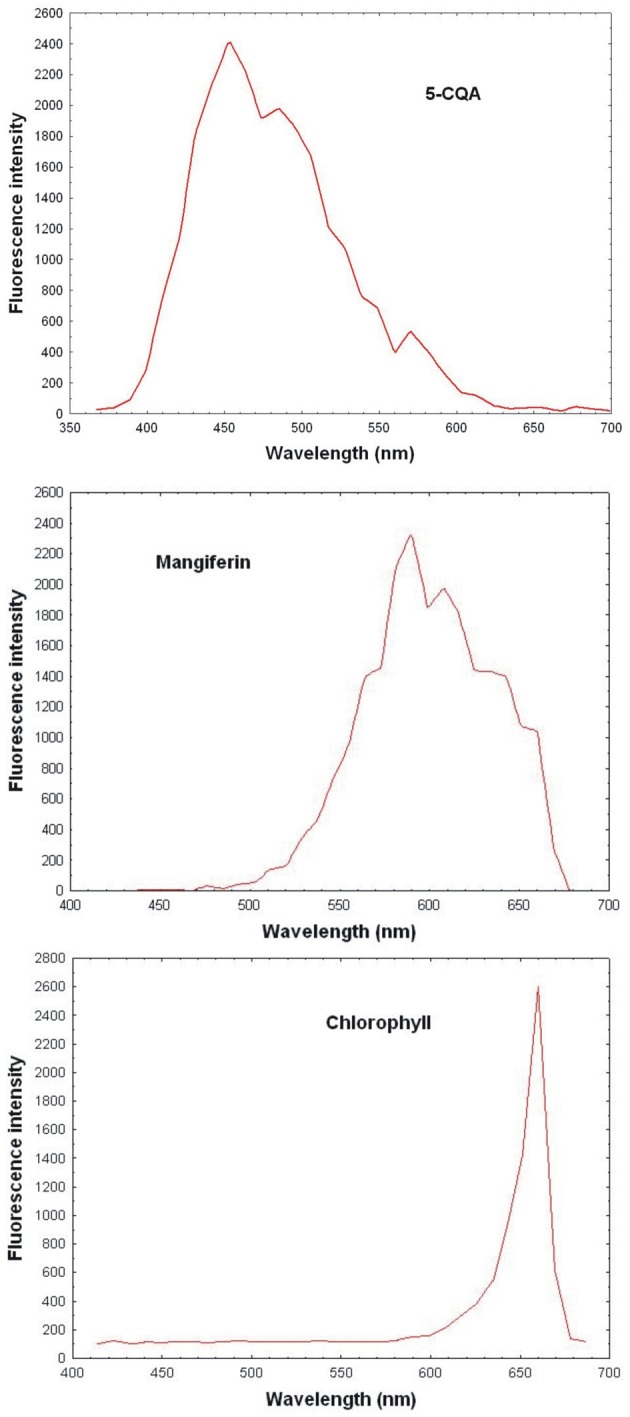
**Reference spectrum of controls (5-CQA, chlorophyll, and mangiferin purified porducts) obtained with 720 nm excitation using multiphoton microscope emission from 365 to 700 nm**.

Reference spectral signatures were acquired on the microscope using controls of products known to be present in mature leaves, i.e., pure 5-CQA and mangiferin powders and chlorophyll extracts. Figure 3 represents the reference spectra of 5-CQA, mangiferin and chlorophyll. Each molecule could be characterized by its absorption and emission spectra. Spectral signatures of 5-CQA, mangiferin, and chlorophyll were then stored in the Spectra Database. The emission spectrum of 5-CQA obtained with the laser system had the same profile and λ_max_ emission (457 nm) as that obtained with a conventional spectofluorometer with a 300 nm excitation wavelength. Spectral signature of chlorophyll presented λ_max_ at 670 nm, as expected. 5-CQA and mangiferin presented a wide spectral range with peaks at about 455 and 590 nm, respectively.

**Figure 4:**
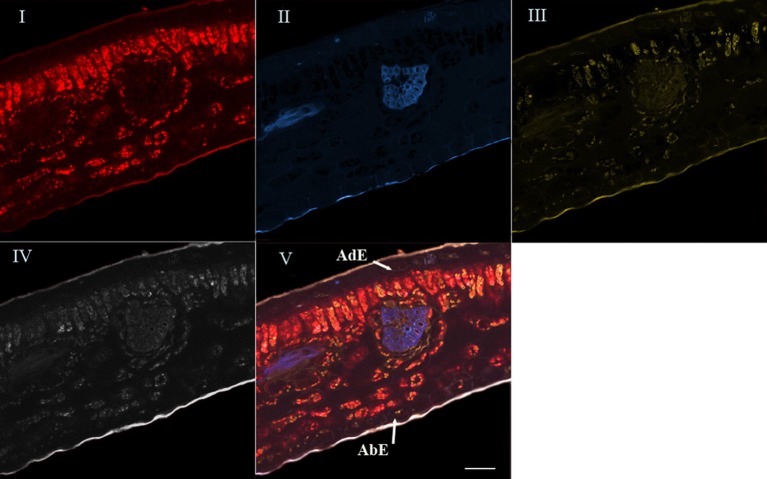
**Spectral analysis by linear unmixing method using chlorophyll, 5-CQA and mangiferin spectra**. Merged image V was splitted into four base images (I, II, III, and IV). Base image I showed histolocalization of chlorophyll, whereas base images (II) and (III) represented histolocalization of 5-CQA and mangiferin, respectively. Finally, base image (IV) depicted other fluorescent compounds (residual fluorescence). AdE, adaxial epidermis; AbE, abaxial epidermis; Scale bar = 50 μm.

For the last step, the advanced linear unmixing process was carried out using chlorophyll, 5-CQA and mangiferin reference spectra (Figure 4). The composite image (V) was generated from four base images (I–IV), corresponding to the localization of chlorophyll (I), 5-CQA (II), mangiferin (III), and residual fluorescence (IV), respectively. Similarity is striking when comparing Figures 2, 4V.

In fact, chlorophyll, 5-CQA and mangiferin spectra looked like ROI1, ROI2, and ROI3 of the Figure 2, respectively. Differences were nevertheless observed due to the fact that several autofluorescent compounds were present at a given pixel. For example, mangiferin spectra did not show a peak at 670 nm, suggesting that ROI3 resulted from both mangiferin and chlorophyll spectra.

High chlorophyll fluorescence was observed in chloroplasts of parenchyma cells (palisade and spongy), but not in the epidermis and leaf veins. 5-CQA fluorescence was mainly distributed in vascular bundles and in the cuticle of the abaxial epidermis, and to a lesser extent in some cells of the adaxial epidermis. It was not detected on the adaxial cuticle. Mangiferin fluorescence appeared in all parenchyma cells and in cuticles (adaxial and abaxial).

### Histochemical structure variations over a time course in ARA leaves

**Figure 5:**
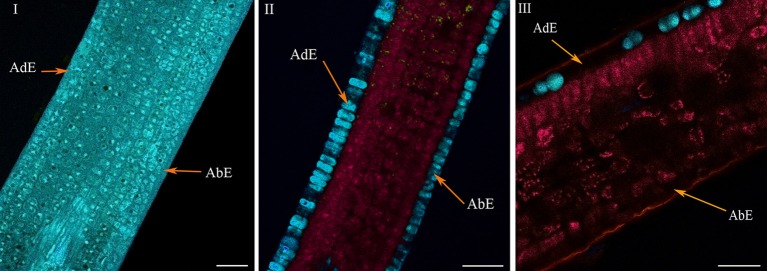
**Histochemical structure of ARA (*C.arabica* cv. Bourbon) leaves at very young (I), young (II), and mature (III) stages**. All these spectral images showed autofluorescence obtained using a multiphoton microscope at 720 nm excitation. AdE, adaxial epidermis; AbE, abaxial epidermis. Scale bar = 50μm.

In very young ARA leaves at the φ2 phase (Lécolier et al., 2009), the classical structure, as seen in Figure 1, was not achieved (Figure 5I) (see also Figure BI in supplementary material). Although the abaxial and adaxial epidermal layers were clearly visible, there was no clear differentiation between palisade and spongy parenchyma. Especially, the absence of clear differentiation characterized the histochemical structure of the leaves. All tissues showed the same blue color in coded autofluorescence (Figure 5I). Only the color intensity varied within leaves, thus highlighting the cell boundaries and vacuoles. Spectral analysis using chlorophyll, 5-CQA and mangiferin spectra showed the presence of chlorophyll at a very low level in the parenchyma (Figure BI, in supplementary material). Moreover, the three spectra did not explain more than 50% of the fluorescence (intense residual fluorescence). Finally, two main differences characterized the histological distribution of 5-CQA and mangiferin: in the case of 5-CQA, the cell limits were not distinguishable, whereas vacuoles clearly appeared. By contrast, mangiferin was absent from cell walls thus highlighting these latter (Figure BI in supplementary material).

The histochemical structure changed very quickly in young leaves over a 4-day period (phase φ3), whereas leaves were always folded at the top of the branch, the classical histochemical structure with two parenchyma (palisade and spongy) became slightly visible (Figure 5II). Advanced linear unmixing confirmed the presence of chlorophyll in parenchyma, thus explaining the red zones observed in autofluorescence (see also Figure BII in supplementary material). The localisation of 5-CQA in both epidermis was also confirmed. Lastly, mangiferin was also present in vacuoles of both epidermal tissues, but at very low intensity. At maturity, the histochemical structure was definitively acquired, with large intercellular spaces in the spongy parenchyma (Figure 5III). The principal effect of age was the lowering of 5-CQA fluorescence in both epidermal layers (Figure BI, in supplementary material).

### Histochemical comparison between some coffea species

**Figure 6:**
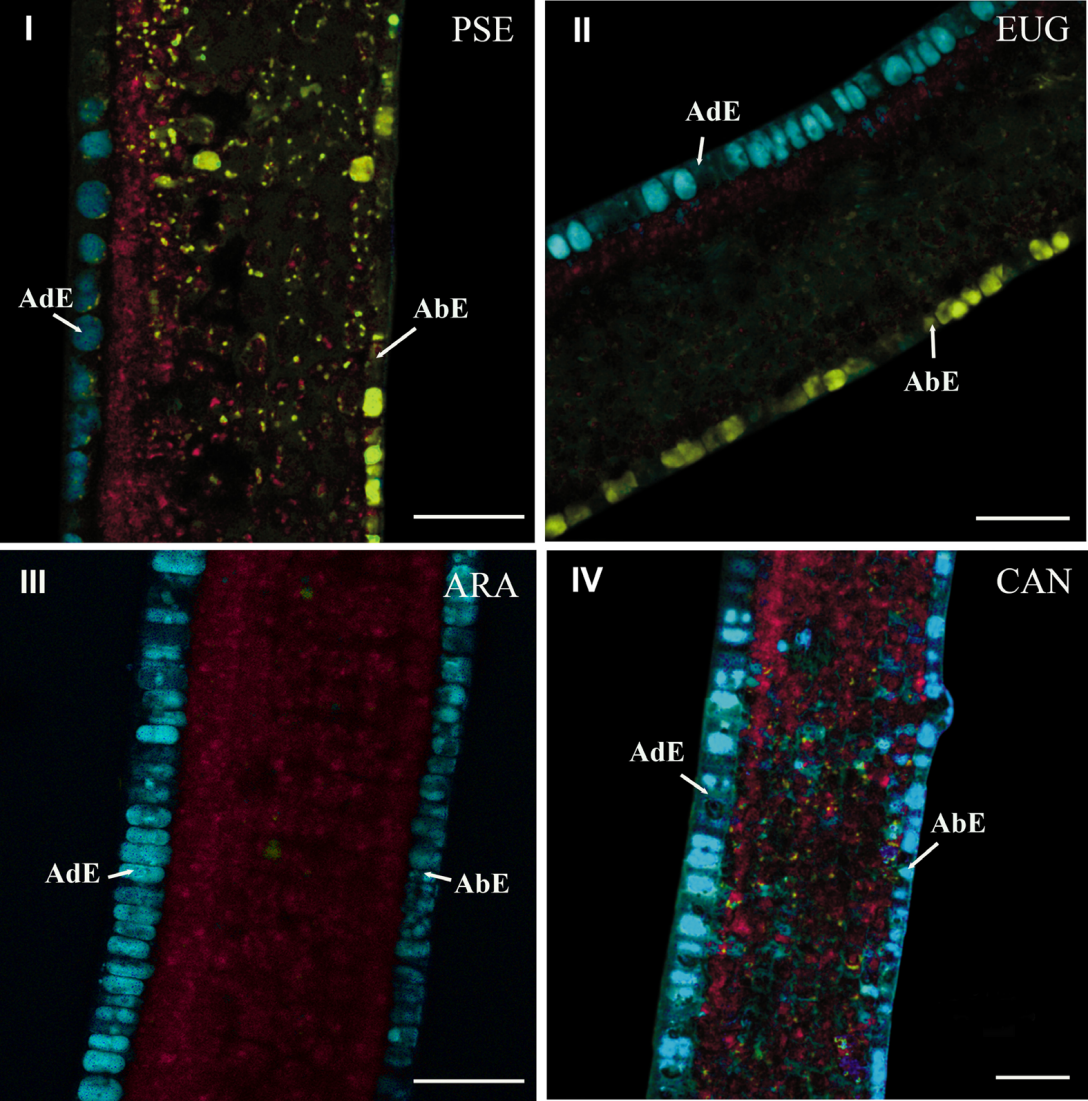
**Cross-sections of young leaves of PSE (*C. pseudozanguebariae*) (I), EUG (*C. eugenioides*) (II), ARA (*C. Arabica* “Bourbon”) (III) and CAN (*C. canephora*) (IV)**. The advanced linear unmixing process was carried out using chlorophyll, 5-CQA and mangiferin reference spectra. AdE, adaxial epidermis; AbE, abaxial epidermis. Scale bar = 50μm.

Histochemical comparison using the advanced linear unmixing process concerned leaves of PSE, EUG, ARA “Bourbon” (ARA) and CAN. In mature leaves, the histochemical structure was similar in the four species (data not shown). This was not the case in young leaves (phase φ3). In this case, two groups of species could be defined, i.e., PSE and EUG vs ARA and CAN. In PSE (Figure 6I) and EUG (Figure 6II), there was strong histochemical differentiation between adaxial and abaxial epidermal tissues at the vacuole level, based on 5-CQA and mangiferin, respectively (Figure 6I). Nevertheless, mangiferin was also present in the spongy parenchyma and, at a lower level, in palisade parenchyma. See also splitted image of PSE leaf in Figure BII (supplementary material). In contrast, ARA (Figure 6III) and CAN (Figure 6IV) did not show mangiferin in adaxial and abaxial epidermal tissues and 5-CQA was strongly present in vacuoles of both of these tissues. In fact, mangiferin was only observable in parenchyma, but as small vesicles.

## Discussion

### Advantages and limits of this new imaging approach

Spectral analysis, combined with the advanced linear unmixing, provides an efficient tool to localize UV-fluorescent metabolites in living plant tissues. Based on the biochemical results obtained on total leaf extracts, i.e., the strong presence of 5-CQA and mangiferin (Campa et al., 2012), the imaging method allowed their spatial localization in mature and young leaves of several *Coffea* species.

The main advantage of this histological approach was the possibility of reaching intact internal tissues within the cross-section. Images were acquired from selected nascent intact cells and tissues far from superficial layers containing damaged and oxidized cells due to cutting. Consequently, and contrary to standard methods using the Neu's reagent, the superficial oxidation of phenols had no effect on the images. The second advantage was the possibility of localizing, at the tissular and cellular level, a particular chemical species characterized by its own emission spectrum. For example, Neu's reagent cannot discriminate between the different types of phenol, whereas the imaging method permitted specific localization of 5-CQA. Moreover, the method does not require any previous extraction of chlorophyll or other pigments. Especially, as mentioned by Zimmermann et al. (2003) and Berg (2004), spectral imaging, combined with advanced linear unmixing, is a powerful method to simultaneously localize several metabolites showing overlapped spectra.

Nevertheless, some caution is required in the interpretation. As any powerful method, the use of spectral imaging combined with linear unmixing has some limits that must be taken in consideration for image interpretation. For example, if metabolites such as isomers or dimers have similar UV-fluorescent spectra, their specific localization is not possible. This was the case for diCQA (dimer of 5-CQA) that had the same spectral signature than 5-CQA. In such situation results coming from biochemical analysis must be considered to support image interpretation. In our study, it was only because quantification by HPLC showed that 5-CQA represented more than 80% of total HQA in mature ARA whose blue zones shown in Figure 4II could mainly reflect the 5-CQA localization.

In fact, the present localization of the 5-CQA by spectral analysis could concern both CQA and diCQA which in young leaves constitutes most of HQA. This problem is also true for classical histochemical analysis using Neu's reagent. Nevertheless, the advantages of spectral analysis, as discussed above, were still relevant. Lastly, the absence of HQA or mangiferin in a tissue or cell was an important result. Indeed, spectral imaging allows detection of very low content. This was the case when visualizing 3-CQA and 4-CQA in Sigma-Aldrich powder of 5-CQA, but also using a dilution gradient (data not shown).

### Practical application of spectral analysis to reveal the histochemical leaf structure and for between species comparisons

The very rapid change in the histochemical leaf structure was the first novel result of the study. In less than 3 days, leaves developed from phase φ2 to phase φ3 (Lécolier et al., 2009) and their histochemical structure changed substantially. Beyond phase φ3, the histological structure was slightly modified and this concerned the spongy parenchyma. In parallel, the blue intensity decreased markedly in both epidermal tissues. This evolution from phase φ3 to leaf maturity has already been observed in CAN using HPLC (Mondolot et al., 2006). In young leaves (phase φ3), 5-CQA and diCQA contents were found to be 2.94 and 2.75% dry matter basis (dmb), respectively, whereas they were 0.58% and 0.14% dmb in mature leaves, respectively.

The differences in 5-CQA and mangiferin histolocalizations between species represented the second novel result of this study. The strong presence of mangiferin in young leaves of PSE was confirmed (Talamond et al., 2008, 2011; Campa et al., 2012). In these leaves (phase φ3), the mangiferin content was 8.6% dmb and decreased up to 5.6% dmb in mature leaves (Campa et al., 2012). Mangiferin has also been localized using UV epifluorescence microscopy (Talamond et al., 2011; Campa et al., 2012). The present results showed a more precise histolocalization of mangiferin in small vesicles of the parenchyma and in vacuoles of the abaxial epidermis, but not in the adaxial epidermis. Conversely, 5-CQA was only localized in the abaxial epidermis. 5-CQA was most abundant and constituted an important reservoir of phenols but its role has not yet been clearly defined. This compound could have at least two different functions, being involved in lignin synthesis, producing the precursor of the lignin structure (guaiacyl and syringyl units), while also being a protective agent against UV or aggressors (Hoffmann et al., 2003). Further experiments are under way to substantiate these assumptions.

The second important result was obtained through the species comparison, highlighting similarities between PSE and EUG, and between CAN and ARA. As EUG and CAN are maternal and paternal parents of ARA (Lashermes et al., 1999), respectively, our work clearly highlighted the paternal origin of the histochemical structure of ARA leaves.

### Conflict of interest statement

The authors declare that the research was conducted in the absence of any commercial or financial relationships that could be construed as a potential conflict of interest.

## Supplementary material

The Supplementary Material for this article can be found online at:

http://www.frontiersin.org/journal/10.3389/fpls.2014.00039/abstract

Figure A **Chemical structures of 5-CQA and mangiferin**.

Figure B **(I)** Spectral analysis by linear unmixing method using 5-CQA, mangiferin and chlorophyll spectra on ARA (*C. Arabica* “Bourbon”) very young leaf splitted image. **(II)** Spectral analysis by linear unmixing method using 5-CQA, mangiferin and chlorophyll on PSE (*C. pseudozanguebariae*) leaf: splitted image.
