# Supplementary figures and images for: Spectral analysis combined with advanced linear unmixing allows for histolocalization of phenolics in leaves of coffee trees
Source: Front Plant Sci. 2014 Feb 18;5:39. doi: 10.3389/fpls.2014.00039 (PMC3927124; doi:10.3389/fpls.2014.00039)

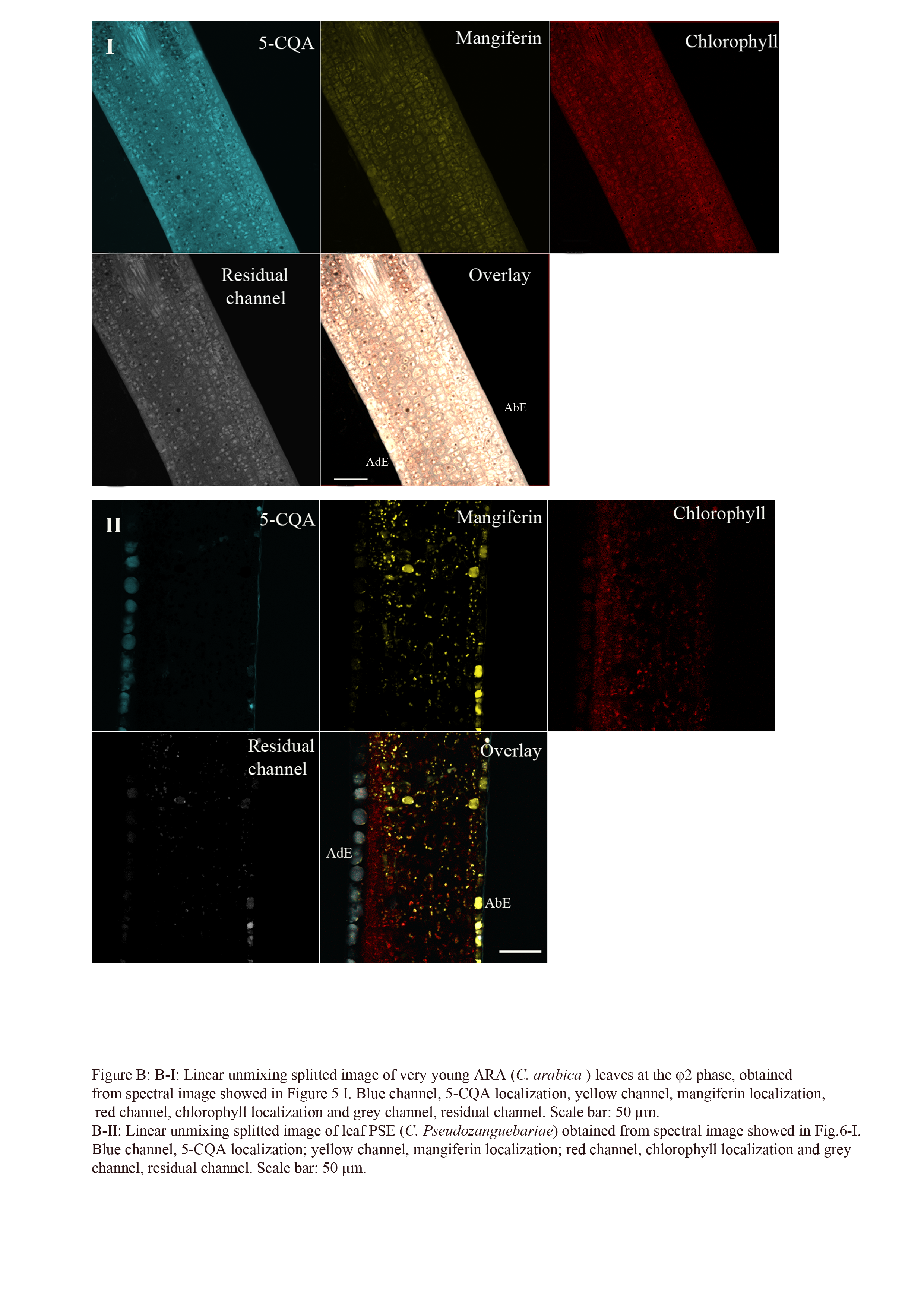

Supplement: Figure A — Chemical structures of 5-CQA and mangiferin. [file Presentation1.ZIP › 76919_Conéjéro_Supplememtary Figure_B.TIF]

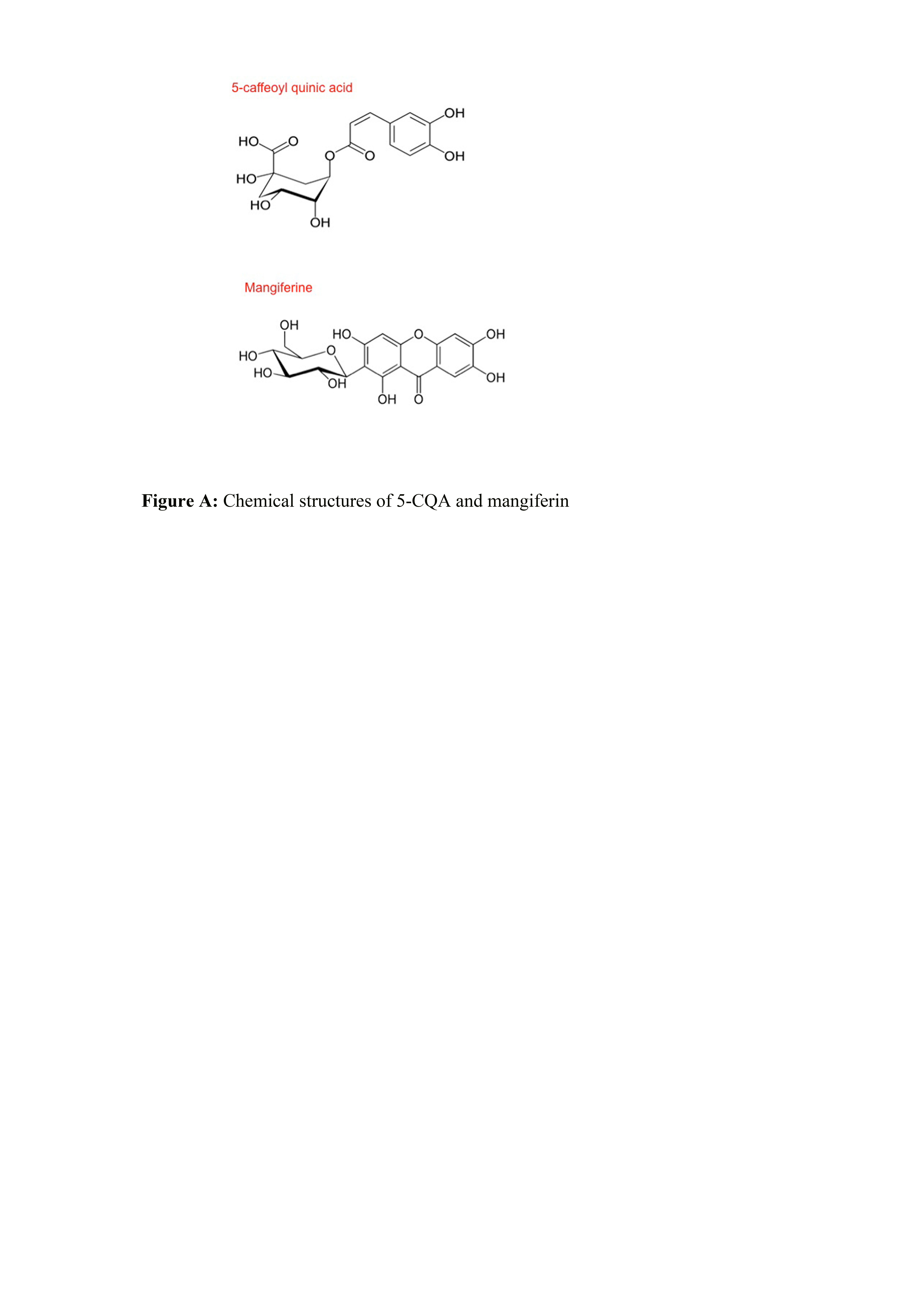

Supplement: Figure A — Chemical structures of 5-CQA and mangiferin. [file Presentation1.ZIP › 76919_Conéjéro_Supplememtary Figure_A.TIF]
